# Primary amenorrhea in a 17-year and 6-month old girl due to celiac disease: A case report

**DOI:** 10.1016/j.amsu.2022.104831

**Published:** 2022-11-05

**Authors:** Ahmad Chreitah, Nour Ibrahim, Mahfoud Eid, Omar Aljanati, Zeina Alkilany, Aria Mohammed, Ibrahim Melhem

**Affiliations:** a Faculty of Human Medicine, Department of Endocrinology Medicine, Tishreen University Hospital, Latakia, Syria; b Department of Endocrinology Medicine, Tishreen University Hospital, Latakia, Syria

**Keywords:** Primary amenorrhea, Celiac disease, Short stature

## Abstract

**Introduction:**

Primary amenorrhea is the absence of the first menarche. There are many causes for primary amenorrhea: gonadal dysgenesis, obstruction of the outflow tract, malformations of the hypothalamus, and the suppression of the hypothalamic pituitary gonadal axis.

**Case presentation:**

A 17-year and 6-month old girl was referred to our Endocrinology Clinic for the evaluation of primary amenorrhea, short stature and reduced general condition. Other than a lack of appetite, there was no past history of any gastrointestinal symptom. Her body mass index was low. The secondary sexual characteristics were at Stage 5 on Tanner score. On investigation, there was no abnormality concerning uterus and ovaries. Celiac disease antibody was highly positive, and the diagnosis of celiac disease was confirmed by small intestinal biopsies. A gluten-free diet was initiated. 4 months later, the patient reached her first menarche.

Celiac disease should be considered among the differential diagnosis of Primary amenorrhea.

Conclusion: healthcare providers should be aware of the extra gastrointestinal manifestations of Celiac disease. Serological testing for Celiac disease should be performed for any patient with primary amenorrhea.

## Highlights

• Amenorrhea (absence of menses) can be a transient, intermittent, or permanent condition.
• Celiac disease (CD) is a systemic autoimmune disease caused by a lifelong intolerance to gluten.
• Malnutrition due to the persistent malabsorption can result in primary amenorrhea.

## 1 Introduction

Celiac disease (CD) is a systemic autoimmune disease caused by a lifelong intolerance to gluten, which causes immunologically mediated inflammatory destruction to the small intestinal mucosa [1].

It affects around 0.7% of the world's population. There is a wide range of clinical manifestations, ranging from malabsorption to asymptomatic patients detected through high-risk group screening [2].

Proof of small intestine villous atrophy in the presence of circulating celiac auto-antibodies and/or a clear response to a gluten-free diet (GFD) is required for diagnosis [2].

CD was originally thought to be a child's disease, but it can affect at any age. And the majority of patients currently have atypical symptoms [2].

The initiation of menses is a complicated process that involves a healthy hypothalamic pituitary gonadal axis (HPG), working ovaries, and a functional outflow tract. One of two criteria can be used to diagnose primary amenorrhea: either no period by the age of 14 in the absence of secondary sexual characteristics such as breast development; or no period by the age of 16 despite normal growth and development with the appearance of secondary sexual characteristics [3].

The useful initial laboratory workup are Follicle-stimulating hormone (FSH), luteinizing hormone (LH), thyroid-stimulating hormone(TSH), and prolactin [4].

Primary amenorrhea can be caused by abnormal HPG axis function or malformation of müllerian structures. Malnutrition due to the persistent malabsorption can disrupt the axis, resulting in primary amenorrhea [5].

The main purpose of the case described here is to report a case of primary amenorrhea caused by celiac disease, which is a very rare association.

This case report has been reported in line with the SCARE criteria 2020 [14].

## 2 Case report

A 17-year and 6-month old girl was referred to our Endocrinology Clinic for the evaluation of primary amenorrhea, short stature and reduced general condition. she issued from a low socio-economic status family. She is the first child of 6 children family. There was no history of chronic diarrhea, vomiting, drug intake or any central nervous symptoms such as headaches or visual disturbances. There was no significant familial medical history. There was no familial history of delayed puberty.

On physical examination she was conscious,very thin, pale with no special facies. Her heart rate was 110 b/m and her blood pressure was 110/65 mm Hg. She attend tanner 5 stage.

Her height was 143 cm (0.1% percentile), her weight was 35 kg (0.1% percentile) and her Body mass index (BMI) was 17.1 kg/m2 (3.3 percentile)**.** There were neither hepatosplenomegaly, nor lymphadenopathy.

Her initial laboratory workup showed severe anemia, a hemoglobin electrophoresis revealed no hemoglobinopathy. We observe values of FSH and LH corresponds to tanner 5, low value of IGF1 and a high value of anti-tissue transglutaminase antibody (Anti TTG IgA).

Her bone age corresponds to 15 years (Greulich & Pyle). An gynecological consultation with *trans*-abdominal sonography of the uterus and ovaries was normal.

Based on the previous results, the patient underwent an upper gastrointestinal endoscopy which showed March3c type on small intestinal biopsies confirming Celiac disease.

A gluten-free diet was advised along with multi-vitamin supplementation: Fe (5mg/kg/day), B9 (1 mg/kg/day) and vitamin D.

The patient was not allowed to eat certain grains that contain gluten such as wheat, barley and foods that contain ingredients made from these grains such as breads, cereals, pastas and Drinks such as beer. We gave her a brochure that includes many foods are free of gluten, including meat, fish, fruits, vegetables, rice, and potatoes. It's acceptable to consume flour produced from beans, potatoes, rice, corn, soy, almonds, cassava, amaranth, quinoa, or corn. These products are provided by several grocery stores and specialized food businesses.

Four months later, the patient's condition significantly improved. she gained weight (3.5Kg), hemoglobin was 9.5 g/100 ml, and she reached her first menarche at the age of 17-year and 10-month.

## 3 Discussion

Primary amenorrhea was a major concern for both the adolescent and her family. Short stature, the obvious weight loss and the presence of severe anemia were not associated with any marked gastrointestinal symptom other than the lack of appetite.

The most important step for the evaluation of primary amenorrhea is to determine the presence of the uterus and ovaries to exclude any outflow tract obstruction. After excluding anatomical and Endocrionological causes with normal values of FSH, LH and in the presence of pallor, thinness with short stature, Celiac disease was suspected with highly positive anti Transglutaminase IGA, which eventually confirmed by the duodenal biopsies.

Tanmahasamut et al. [6], and Kriplani et al. [7], didn't find any primary amenorrhea caused by gastrointestinal causes. Subbiah et al. [8], reported a 17-year-old girl with primary amenorrhea diagnosed with hypothyroidism. According to Ben Nsir et al. [9], a 17-year-old girl with primary amenorrhea, was diagnosed with a Xanthogranuloma, which is extremely rare in pediatric population.

Despite CD is a widespread disease but it is an uncommon cause of primary amenorrhea and there were very few case reports. Costa et al. [10], and Pradhan et al. [11], presented two cases with primary amenorrhea and lately diagnosed with CD.

Most extra intestinal symptoms of CD, such as short stature, infertility, and anemia, have been proven to improve with a GFD [2].

Chronic diseases,lifestyle factors such as stress, and excessive exercise may cause HPG axis suppression. As an adaptive reaction to long term metabolic energy insufficiency, hypogonadotropic hypogonadism and functional hypothalamic amenorrhea occur.

If LH and FSH are low or average along with a history of severe weight loss, feeding problems or stress, a hypothalamic amenorrhea is likely. Menstrual cycle problems may accompany a systemic disease when it is severe enough to disrupt hypothalamic GnRH secretion and/or when it is linked to nutritional shortages (as primary amenorrhea).

Essential nutrient deficiencies can have a negative impact on fertility (as delayed menarche and early menopause) [12].

With time classical symptoms of CD have changed dramatically from a full-blown symptomatic form with distended abdomen, diarrhea, weight loss and general malaise to a major group with mild non-specific symptoms [1]. These symptoms mainly include long-lasting extra intestinal features as anemia, osteoporosis and growth failure [13].

The exact mechanisms by which celiac disease results in these changes are unclear. However, hormonal factors and related nutritional factors have been implicated [1].

Despite the wide prevalence of CD in the Middle East and the current situations in Syria, we did not meet until now a such advanced case of CD with late diagnosis.

CD could be considered among the differential diagnosis of Primary amenorrhea as just a GFD can manage.

## 4 Conclusion

The uniqueness of the case is describing an unrecognized relationship between primary amenorrhea and celiac disease. Primary amenorrhea can be caused by celiac disease, which is a rare but treatable cause. Healthcare providers should be aware of the extra gastrointestinal manifestations of CD to ensure timely screening and early Initiation of a GFD. We suggest performing serological testing for Celiac disease for any patient with primary amenorrhea without a clear cause.

Table 1 showed iron deficiency anemia.

**Table 1:** Biochemical laboratory workup.

Test	Result	Normal values	Test	Result	Normal values
HgB	7.5	More than 11 g/100 ml	AST	35	0–35 U/L
MCV	57	80-95 FL	Creatinine	0.7	0.5–1.1 mg/dl
Iron	40	60–160 μg/100 ml	Urea	15	10–20 mg/dl
ALT	30	4–36U/L			

**HgB:** Hemoglobin, **MCV:** Mean corpuscular volume, **ALT:** Alanine Aminotransferase, **AST:** Aspartate Aminotransferase.

Table 2 showed a high value of anti TTG IGA and low value of IGF1 may be related to her low BMI.

**Table 2:** Hormonal laboratory workup.

Test	Result	Normal values	Test	Result	Normal values
Cortisol	21	2–25μg/dl	Prolactin	14	0–20ng/mL
FSH	2.2	a	anti TTG IGA	230	7–10 U/ml
LH	2.7	a	IGF1	84	149–509ng/ml
TSH	3	0.5 TO 5 μU/mL			

**FSH:** follicle stimulating hormone, **LH:** luteinizing hormone, **TSH:** thyroid stimulating hormone, **anti TTG IGA:** anti tissue transglutaminase, **IGF1:** Insulin-like Growth factor 1.

a The values of FSH and LH corresponds to tanner 5.

## Sources of funding

This research did not receive any specific grant from funding agencies in the public, commercial, or not-for-profit sectors.

## Ethical approval

This case report did not require review by the Ethics Committee Tishreen university hospital, Latakia, Syria.

## Consent

Written informed consent was obtained from the patient's parents for publication of this case report and accompanying images. A copy of the written consent is available for review by the Editor-in-Chief of this journal on request.

## Author contributions

Ahmad Chreitah: contributed in data interpretation, and as a mentor and reviewer for this case report.

Nour Ibrahim: contributed in performing an extensive literature review.

Mahfoud Eid: contributed as a mentor and reviewer for this case report.

Omar Aljanati: contributed in writing the paper.

Zeina alkilany: contributed in writing the paper.

Aria Mohammed: contributed in writing the paper.

Ibrahim Melhem: contributed in writing the paper.

## Registration of research studies

Not applicable.

## Guarantor

Mahfoud Eid.

## Annals of medicine and surgery

The following information is required for submission. Please note that failure to respond to these questions/statements will mean your submission will be returned. If you have nothing to declare in any of these categories then this should be stated.

## Provenance and peer review

Not commissioned, externally peer reviewed.

## Declaration of competing interest

All of the authors declare that they have no competing interests.
